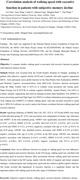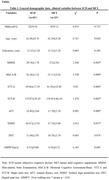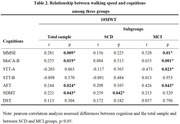# Correlation analysis of walking speed with executive function in patients with subjective memory decline

**DOI:** 10.1002/alz.084280

**Published:** 2025-01-03

**Authors:** Lei Chen, Jiayu Wang, Linlin Li, Qiansen Feng, Ziqi Wang, Mingjun Duan

**Affiliations:** ^1^ Zunyi Medical University, Zunyi, Guizhou China; ^2^ Zunyi Medical University, Zunyi, GuiZhou China; ^3^ Department of Geriatrics, the Fourth People’s Hospital of Chengdu, ChengDu China; ^4^ The Clinical Hospital of Chengdu Brain Science Institute, MOE Key Lab for Neuroinformation, School of Life Science and Technology, University of Electronic Science and Technology of China, Chengdu, Sichuan China; ^5^ The Clinical Hospital of Chengdu Brain Science Institute, MOE Key Lab for Neuroinformation, School of Life Science and Technology, University of Electronic Science and Technology of China, ChengDu China

## Abstract

**Background:**

To examine whether walking speed is associated with executive function in patients with subjective memory decline.

**Method:**

Patients were recruited from the Fourth People’s Hospital of Chengdu, including 63 patients with subjective cognitive decline (SCD) and 23 patients with mild cognitive impairment (MCI). Each participant assess global function by the Mini‐Mental State Examination (MMSE) and Montreal Cognitive Assessment‐Basic Scale (MoCA‐B). Executive function assessments using the Shape Symbol Task A (STT‐A) to evaluate visual perception and writing speed, Shape‐Tracing Test B (STT‐B) to evaluate cognitive flexibility. Animal Fluency Test (AFT) to evaluate semantic organization and retrieval strategies, Symbol Digit Modalities Test (SDMT) to assess processing speed and Digit Span Test (DST) to evaluate information processing processes. 10‐m walking test (10MWT) to evaluate walking speed, with time recorded converted to speed (m/s). SPSS 26.0 software was used to analyze the Pearson correlation between walking speed and executive function.

**Result:**

Significant difference of MMSE, MoCA‐B, AFT, STT‐A, STT‐B and SDMT were found in both individual groups (P <0.05), and associations were independent of Gender, Age, Education and 10MWT. In the total combined group, 10MWT were significantly associated with Education (r = 0.267, p = 0.014), MMSE (r = 0.281, p = 0.009), MoCA‐B (r = 0.255, p = 0.019), AFT (r = 0.244, p = 0.024) and SDMT (r = 0.221, p = 0.043), negatively correlated with Age (r = ‐0.267, p = 0.013). In the SCD group, 10WMT were identified positive association with SDMT (r = 0.259, p = 0.042), negative correlation with Age (r = ‐0.282, p = 0.026). In the MCI group, 10WMT were identified positive association with Education (r = 0.655, p = 0.001), MMSE (r = 0.528, p = 0.01), MoCA‐B (r = 0.655, p = 0.001), AFT (r = 0.426, p = 0.043), negative correlation with STT‐A (r = ‐0.473, p = 0.023).

**Conclusion:**

There was no difference between two groups in walking speed, but were differences in global cognition and executive functions. In the SCD group, walking speed was correlated with processing speed in executive function. More associations between walking speed and executive function were found in the MCI group, mainly with the ability of organize and extract semantic strategies, visual perception and writing motor speed and also related to global cognitive function. The results revealed that impairment of executive function is a significant feature and has important relation with walking speed in the early stages of Alzheimer’s disease.